# Insight on thermal stability of magnetite magnetosomes: implications for the fossil record and biotechnology

**DOI:** 10.1038/s41598-020-63531-5

**Published:** 2020-04-21

**Authors:** Jefferson Cypriano, Mounib Bahri, Kassiogé Dembelé, Walid Baaziz, Pedro Leão, Dennis A. Bazylinski, Fernanda Abreu, Ovidiu Ersen, Marcos Farina, Jacques Werckmann

**Affiliations:** 1 Instituto de Microbiologia Paulo de Góes, Universidade Federal do Rio de Janeiro, 21941-902, Rio de Janeiro, Brazil; 2 Institut de Physique et Chimie des Matériaux de Strasbourg (IPCMS), UMR 7504 CNRS-Université de Strasbourg, 23 rue du Loess, 67034 Strasbourg, France; 3 School of Life Sciences, University of Nevada at Las Vegas, Las Vegas, 89154-4004 USA; 4 Instituto de Ciências Biomédicas, Universidade Federal do Rio de Janeiro, 21941-902, Rio de Janeiro, Brazil; 5 Centro Brasileiro de Pesquisas Físicas, LABNANO, rua Xavier Sigaud, 150, CEP, 22290-180 Rio de Janeiro, Brazil; 6 Fritz-Haber-Institut der Max-Planck-Gesellschaft, Department of Inorganic Chemistry, Faradayweg 4-6, 14195 Berlin, Germany

**Keywords:** Biomaterials, Nanobiotechnology

## Abstract

Magnetosomes are intracellular magnetic nanocrystals composed of magnetite (Fe_3_O_4_) or greigite (Fe_3_S_4_), enveloped by a lipid bilayer membrane, produced by magnetotactic bacteria. Because of the stability of these structures in certain environments after cell death and lysis, magnetosome magnetite crystals contribute to the magnetization of sediments as well as providing a fossil record of ancient microbial ecosystems. The persistence or changes of the chemical and magnetic features of magnetosomes under certain conditions in different environments are important factors in biotechnology and paleomagnetism. Here we evaluated the thermal stability of magnetosomes in a temperature range between 150 and 500 °C subjected to oxidizing conditions by using *in situ* scanning transmission electron microscopy. Results showed that magnetosomes are stable and structurally and chemically unaffected at temperatures up to 300 °C. Interestingly, the membrane of magnetosomes was still observable after heating the samples to 300 °C. When heated between 300 °C and 500 °C cavity formation in the crystals was observed most probably associated to the partial transformation of magnetite into maghemite due to the Kirkendall effect at the nanoscale. This study provides some insight into the stability of magnetosomes in specific environments over geological periods and offers novel tools to investigate biogenic nanomaterials.

## Introduction

Magnetosomes are intracellular single domain nanocrystals of magnetite (Fe_3_O_4_) or greigite (Fe_3_S_4_) enveloped by a lipid bilayer. These structures are produced by magnetotactic bacteria through a genetically controlled biomineralization process^1^. Besides having control over the chemical composition, magnetic properties, shape and size of magnetosome crystals, magnetotactic bacteria also have a cytoskeletal-like structure along which magnetosomes are organized in single or multiple chains, imparting to the cell a magnetic moment and the ability to passively orient along magnetic field lines, in particular the Earth’s geomagnetic field^2^. Currently magnetotactic bacteria are affiliated to several groups in the domain *Bacteria* and may have existed since the Archean Eon^3^. The ecological importance of this apparently ancient trait is to assist bacterial chemotaxis in more efficiently locating an optimal position in vertical chemical gradients for survival using the geomagnetic field^1,3^.

Specific properties of magnetosome magnetite crystals have long been used to differentiate fossilized remains of magnetotactic bacteria (magnetofossils) in sediments and rocks from magnetites of inorganic origin^4^. In some cases, magnetofossils appear to remain preserved for millions of years^5^. The resistance of magnetofossils to diagenesis and extreme environmental changes has been described in some detail^6^. Thus, these nanoparticles may provide an important record of ancient ecosystems.

Several techniques are used to determine the presence of biogenic magnetite in sediment^6–8^. These include characterizations of crystal sizes and shape distributions, crystal morphologies, arrangement, chemical purity, and crystallographic perfection determined using high-resolution transmission electron microscopy (HRTEM), off axis electron holography and nanometer scale chemical analysis^9–11^. To our knowledge, a real-time analysis of the thermal stability of magnetite magnetosomes in an oxidative environment has never been performed. This information is essential in understanding magnetofossil stability in the environment and in any prediction of modification of their crystalline structure over geological time scales and extreme conditions.

 Characterization of the thermal stability of magnetosomes under oxidizing conditions is also relevant from a technological point of view. The unique properties of magnetosomes described above, especially the presence of an external lipid bilayer with associated proteins, place them in the spotlight as tools in the next generation technologies^12^. In nanomedicine, for example, magnetite magnetosomes have proven to be efficient tools in the development of both drug delivery systems and in magnetic fluid hyperthermia^13,14^. Enzymatic nanocomplexes for industrial applications have also been developed using magnetite magnetosomes. In these cases, enzymes can be attached to the surface of magnetosomes to concentrate or eliminate target molecules^15,16^. For both biomedical and industrial applications, the magnetic properties, as well as morphological and structural features of magnetosomes, should be maintained during the treatment period or as much as possible for reuse of enzymatic complexes, respectively. So far, the studies reporting the modification of magnetite magnetosomes properties when subjected to thermal treatments were only performed under relatively moderate temperatures, which are relevant for biomedical applications that usually reach values below 50 °C; in this general framework, it is worthy to note that a nanometer-scale characterization of the modifications of these structures induced by a heat induction process was not reported until now^17^.

Several studies have shown that inorganic magnetite is transformed into maghemite at moderate temperatures (below 250 °C) under an air atmosphere^18–21^. Here we subjected elongated prismatic magnetite magnetosomes to temperatures ranging from 150 to 500 °C under O_2_ at atmospheric pressure and analysed the shape, oxidation state variation and crystallographic structure of the magnetosomes by high-resolution, tomographic and analytical electron microscopy techniques.

## Results

### Oxidation from 150 °C to 300 °C

**Figure 1:**
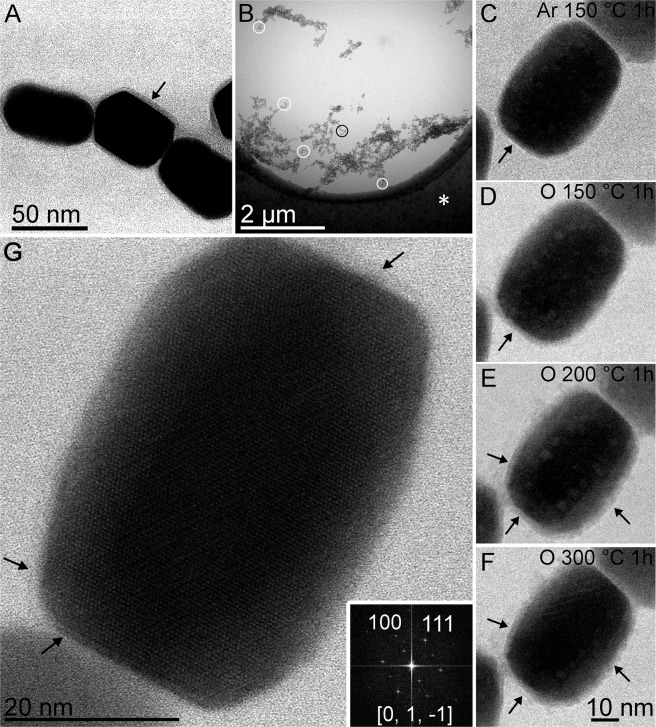
Evaluation of the thermostability of magnetite magnetosomes by bright field scanning transmission electron microscopy (BF-STEM). (**A**) Purified magnetosomes showing the presence of the surrounding membrane (arrow). (**B**) Low magnification image of the E-chip used for the environmental gas STEM analysis (asterisk) showing the analysed areas in the “electron transparent” wheel of the E-chip device (circles). The black circle shows the region exhibited at higher magnification in (**C-F**) where the same magnetosomes subjected to different conditions are shown. White circles correspond to areas used in other analysis. (**C**) Magnetosomes subjected to 150 °C for 1 h under an argon (Ar) atmosphere. (**D**) Magnetosomes subjected to 150 °C for 1 h under an O_2_ atmosphere. (**E**) Magnetosomes subjected to 200 °C for 1 h under an O_2_ atmosphere. (**F**) Magnetosomes subjected to 300 °C for 1 h under an O_2_ atmosphere. (**G**) High-resolution-TEM image of the same magnetosome after 300 °C exposed to O_2_, showing the crystal structure without alteration and the presence of a membrane (arrows). The arrows in (**C-F**) were included for comparison of the thicknesses of the magnetosome membranes. Images show that membranes are apparent in (**D-F**) and present a non-uniform aspect like exfoliation around the magnetite crystal (arrows). Scale bar in (**F**) applies to (**C-E**).

**Figure 2:**
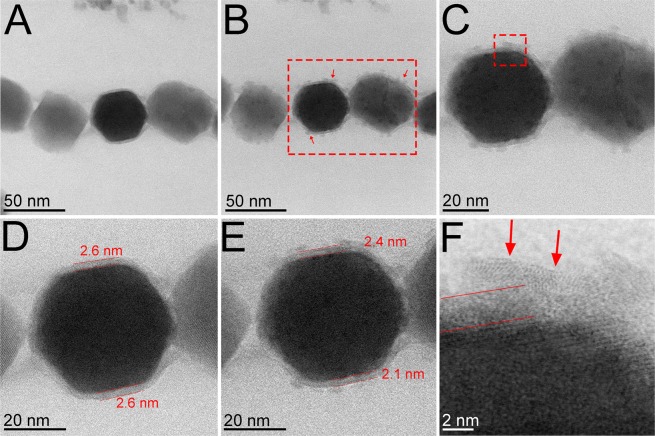
BF-STEM images before and after oxidation treatment at 300 °C. Observation made at room temperature, without the upper membrane which closes the E-Chip containing the sample. This greatly increase the signal-to-noise ratio in the image, due to the absence of the interaction of the electrons with silicon nitride cover and with gaseous oxygen atoms. (**A**,**D**) Before oxidation treatment, the initial thickness of the membrane is of about 2.6 nm. (**B**,**C**) After oxidation treatment, the thickness of the membrane is reduced (arrows in B). (**E,F**) show details of the thickness (E) and of the structure (arrows in F) of the membrane after treatment.

Conventional transmission electron microscopy (CTEM) images of the purified magnetite magnetosomes of *Magnetovibrio blakemorei *strain MV-1 showed the presence of the magnetosome membrane, which envelops each crystal (Fig. 1A). Deposition of the magnetosomes on the *in situ *E-chip was checked and the areas for study were selected (Fig. 1B; circles). For real-time thermal stability evaluation of magnetosomes, the sample was heated to 150 °C under an Ar atmosphere for 1 h and no changes were observed in the magnetite crystal or magnetosome membrane (Fig. 1C; arrow). The magnetosome membrane became more apparent upon the injection of O_2_ in the *in situ* chamber and increasing the temperature to 300 °C. The continuous temperature increase (150–300 °C) also affected the smooth appearance of the magnetosomes membrane, which became more irregular and rough, suggesting an aggregation of lipids and denaturation of proteins (Fig. 1D-F, arrows). The comparison of the crystalline structure of magnetosomes before and after being subjected to heat and an oxidizing atmosphere showed no changes in the mineral component of the magnetosomes. Although ultrastructural changes were observed on the magnetosome membrane after heating the sample to 300 °C under oxidizing conditions (Fig. 1C–G), in order to obtain a better evaluation of the membrane transformation, observations of the same field were made at room temperature without the upper nitride silicon window of the holder’s chamber (Fig. 2), which allows obtaining images of better resolutions, unaffected by the interactions of the electrons with the upper membrane and the oxygen present in the Protochips cell.

High-resolution bright-field scanning transmission electron microscopy (BF-STEM) observation after cooling showed the presence of the membrane around the magnetite crystals (Fig. 2), suggesting that the bilayer deforms and undergoes organizational changes after heating. Comparison of the structure of the membrane before (Fig. 2A,D, Supplementary Fig. S1) and after oxidation (Fig. 2B,C,E,F), clearly shows that the structure formed in the course of the oxidation, which surrounds it under the appearance of a hair or plum, does not seem to have affected its original morphology. Its thickness is reduced as shown by the comparison between the images in Fig. 2D,E, but entirely envelopes the crystals.

**Figure 3:**
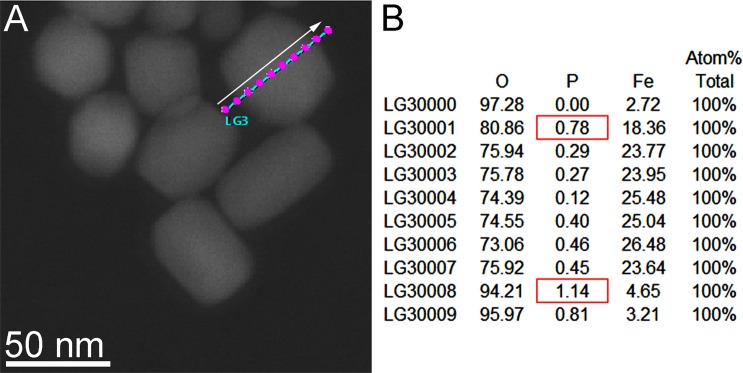
High-resolution STEM images and energy-dispersive X-ray spectroscopy (EDS) line scan microanalysis of magnetosomes heated to 300 °C. (**A**) HAADF image of isolated magnetosomes showing the EDS line scan region (blue line; pink dots represent areas corresponding to measurements displayed in (**B**); the white arrow represents the direction of scanning); (**B**) Line scan results showing that phosphorus is detected over the entire crystal, and this signal increases close the facets (red rectangles, because the electron beam passes through a larger thickness of membrane, in fact the beam close to the edges has a parallel direction to the membrane. Note that minor amount of iron is detected outside the magnetosome (at the LG30000 and LG30009 positions). A small signal of Fe could orginate from the polepiece of the microscope, as related by Williams and Carter^28^.

**Figure 4:**
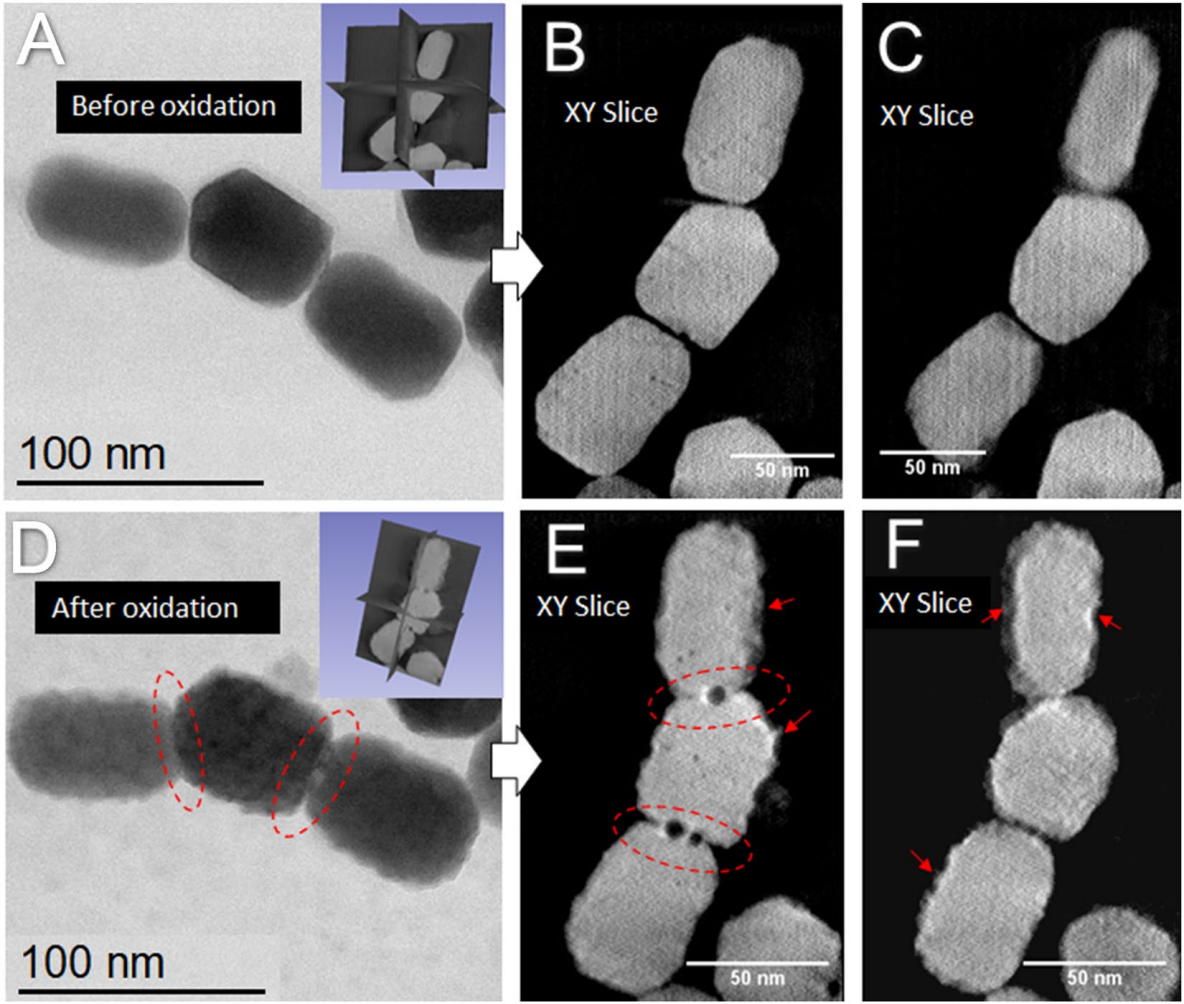
STEM image and tomography slices of a magnetosome chain before and after oxidation at 300 °C. Before: (**A**) BF-STEM image. Tomography performed in HAADF-STEM mode from −55.5° to +55.5° with a step of 1.5° and acquisition magnification of 800.000X. (**B**,**C**) Slices extracted from the reconstructed volume at two different depths. After: (**D**) BF-STEM image. Tomography performed in the same conditions as previously. (**E**) and (**F**) Slices extracted from the reconstructed volume at two different depths. The dotted ellipses in red surround nanopores formed at the interfaces between magnetite nanocrystals. The arrows point to the presence of a significant roughness at the external faces of the magnetosome.

Energy-dispersive X-ray spectroscopy (EDS) line scan analysis after sample heating and cooling corroborated this result by detecting phosphorus across the magnetosome (Fig. 3A,B). Interestingly, high amount of iron is only detected until the edge of the magnetosome crystal and not in the regions surrounding the crystals, suggesting that the magnetite crystals were not degraded during the increase in temperature and exposure to O_2_. However, in the analysis approach using STEM tomography before and after oxidation (Fig. 4 and Supplementary Fig. S2), nanopores formed at the interfaces between the magnetite nanocrystals and the presence of a significant roughness of the outer faces of the crystals were observed after treatment (Fig. 4E,F).

### Oxidation from 300 °C to 500 °C

**Figure 5:**
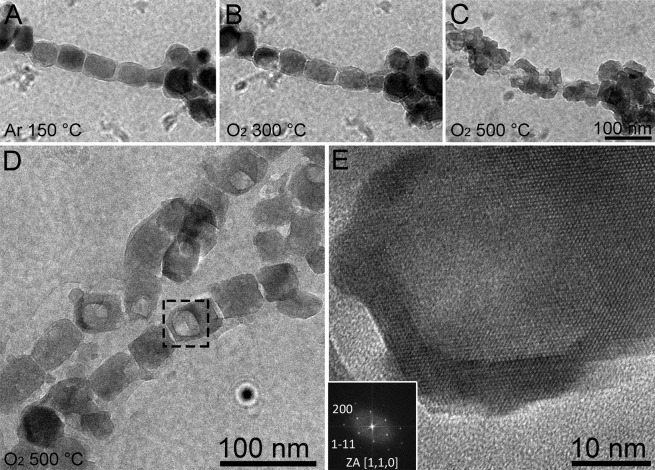
CTEM images of magnetosomes during *in situ* heating experiment when subjected to 150 °C until 500 °C and exposed to Ar and O_2_. From (**A**) to (**C**) in irradiation condition, (**A**) CTEM image of a chain of magnetosomes at 150 °C with Ar; (**B**) CTEM image of the same chain shown in (**A**) after heating to 300 °C and exposed to O_2_; (**C**) CTEM image of the same chain shown in (**B**) after heating to 500 °C and exposed to O_2 _and irradiated by the electron beam; (**D**) CTEM image of magnetosomes at 500 °C exposed to O_2_ obtained in a region that has not been irradiated by the electron beam. Note that the general shape of the magnetosome is maintained, but cavities occur in some crystals. (**E**) Enlarged image of the boxed area in Fig. D (see also the supplementary Fig. S3) showing a cavity in the magnetosome structure. Inset shows the FFT corresponding to this high-resolution image. The corresponding zone axis can be assigned to magnetite or maghemite but also to a mixed structure (see also Supplementary Fig. S5).

**Figure 6:**
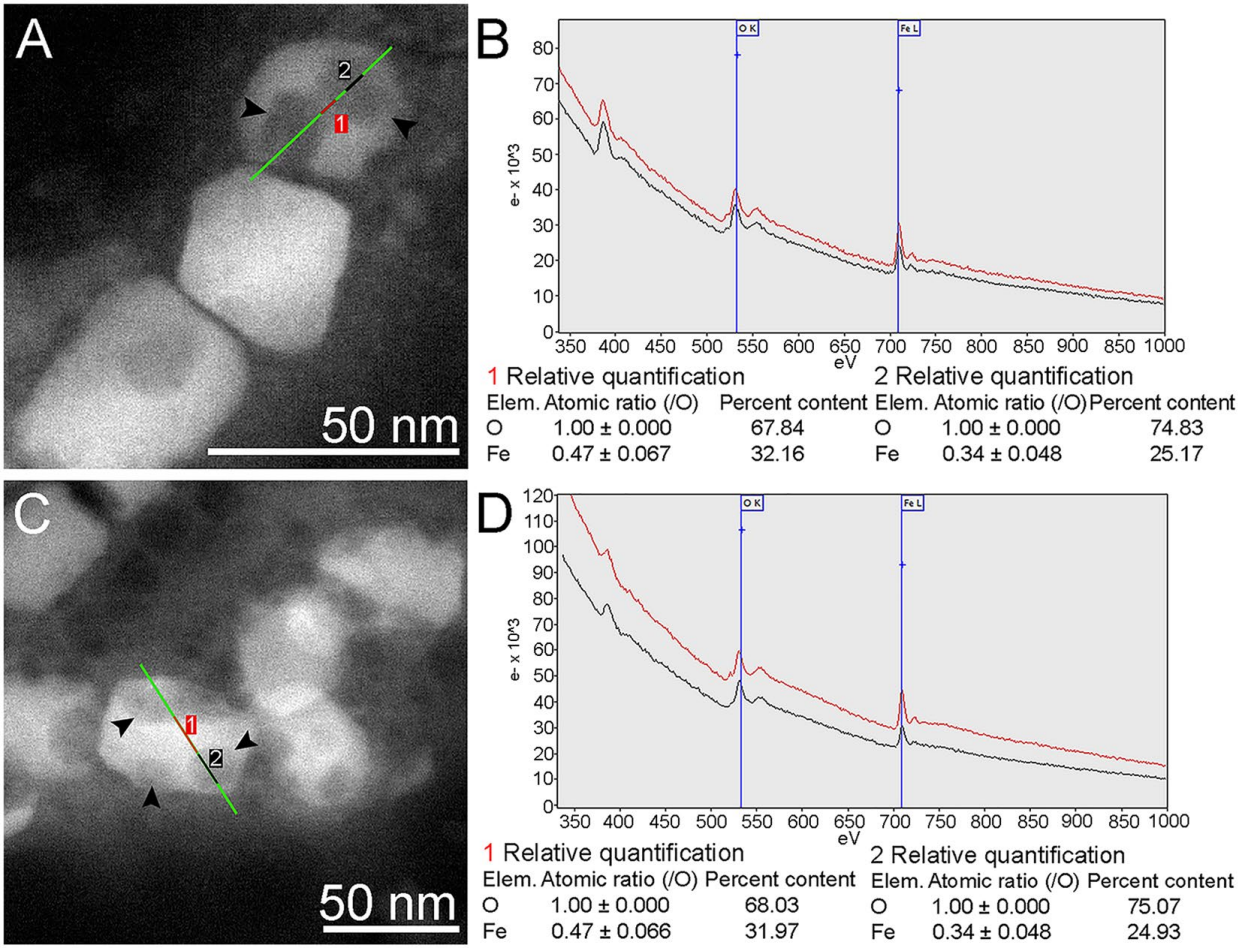
HAADF-STEM images and EELS microanalysis of magnetosomes after heating until 500 °C and exposure to O_2_, showing the irregular contrast on magnetosomes and the presence of large cavities. (**A**) HAADF-STEM image of magnetosomes with cavities. Non-altered regions represented by the red line segment (number 1) and a cavity indicated by the black line segment (number 2) were selected for EELS analysis showed in (**B**); (**B**) EELS spectra and relative quantification of regions indicated by red line 1 and black line 2 in (**A**) for oxygen 540 eV and iron 710 eV; (**C**) HAADF-STEM image of magnetosomes with cavities. Non-altered regions represented by the red line (number 1) and a cavity indicated by the black line (number 2) were selected for EELS analysis showed in (**D**); (**D**) EELS spectra and relative quantification of regions indicated by red line segment 1 and black line segment 2 in (**C**) for oxygen 540 eV and iron 710 eV.

When magnetosomes were heated until 500 °C significant alterations of the magnetite crystals were observed (Fig. 5). Although in CTEM magnetosomes seemed completely damaged (Fig. 5C), in the non-irradiated regions illustrated inFig. 5D,E the general morphology of the crystals was preserved. However, a hollow structure, similar to a cavity, appears in the magnetosomes. Similar structures were observed in the experiment with the microscope operating in STEM mode (Fig. 6A,C). Semi quantitative analysis of the damaged areas of theses magnetosomes by ELNES (energy loss near edge structure) of oxygen (O-K edge around 540 eV) and iron (Fe-L edge around 710 eV) indicated modifications in their chemical composition (Fig. 6). Bright areas in the HAADF images of Fig. 6 represent the unaffected portion of magnetosomes, while dark areas are cavities that presented significant iron loss and unbalance proportion of oxygen in relation to magnetite, showing a change in the mineral phase (Fig. 6B,D). The presence of iron oxides was not detected in the area around the magnetosome in these samples, by using EELS (see also Supplementary Fig. S4).

## Discussion

Based on our CTEM, BF-STEM high-angle annular dark field (HAADF-STEM) mode, EDS and electron energy loss spectroscopy (EELS) results, magnetosomes are thermostable nanoparticles when subjected to temperatures up to 300 °C. It is difficult to clearly visualize the morphology of the membrane from a direct analysis of the images (Fig. 1) obtained at different temperatures 150 °C, 200 °C and 300 °C. Indeed, in the environmental TEM mode, the images of the magnetosomes are obtained by collecting the transmitted electron beam which cross also the double SiN membrane (i.e., the top and bottom chips which isolate the environmental cell) and that degrades the contrast of the images due to the additional diffusion contribution; however, it is possible to distinguish some changes at 200 °C and 300 °C which are highlighted by arrows in Fig. 1E,F. These changes can be better visualized after the return to the room temperature and the removal of the sealing cover (Fig. 2). Using these specific observation conditions, some details of the microstructure of the sample after treatment can be clearly observed, particularly the fact that the general appearance of the structure can be assigned to a hair or plume which seems to originate from the membrane and the surrounding part of the magnetosome (Fig. 2B,C,E,F). From a quantitative point of view, though the membrane keeps its initial appearance, its thickness goes from a value of about 2.6 nm to values between 2.1 and 2.4 nm.

The membrane of magnetosomes is a lipid bilayer functionalized by specific proteins involved in the synthesis of magnetosome. Most of the membrane lipids exist in the lamellar or bilayer phase, in particular in a lamellar liquid crystalline phase but sometimes in the lamellar gel phase^22^. Until now we were not able to specifically determine the structure and physical properties of lipid bilayer membranes of magnetosomes. However, it is generally admitted that lipid bilayer membranes may exhibit, in some cases, a typical behavior specific to a gel or to a liquid, depending on the temperature. It should be thus assumed that, with the rise in temperature, the magnetosome membrane passes through a liquid state which leads to the segregation of the proteins on their surface. This state is a posteriori visible in the electron microscopy images through the formation of plumes associated to a subsequent decrease of the thickness. However, it is important to note that the general morphology and the global localization of membranes around the crystals are preserved during the thermal treatment.

In nature, the oxidation of magnetite to maghemite is probably the most common oxide mineral alteration. This reaction invariably takes place in fine particles after exposure to air at room temperature. The oxidation of magnetite to maghemite at temperatures close to 200 °C was reported when the magnetite is well ordered and free of stacking faults, whereas magnetite containing stacking faults oxidizes partially to α-Fe_2_O_3 _and lowered the transformation of magnetite to hematite^19–21^. As shown in the high-resolution image (Fig. 1G) the magnetite crystalline structure was conserved at the subnanometric scale when samples were subjected to 300 °C in the presence of O_2_. No defects were visible (phase transformation corrosion recrystallization or dislocations) and the perfection of the crystalline structure characteristic of biogenic magnetite, was preserved. The STEM mode can be considered to a certain extent as a method of chemical analysis because the contrast of the images varies roughly as the square of the mass of the studied sample, being thus sensitive to its thickness and to the mass of the atoms present in the sample^23^. Combined with tomography, this technique allows a three-dimensional view of the nanocrystals with a qualitative analytical selectivity. The extracted sections are the result of a deconvolution and reconstruction process of hundreds of images and consequently the signal-to-noise ratio in the sections extracted from the tomograms is considerably improved as compared to classical 2D images; in addition, this “slice by slice” analysis allows for a fine analysis of the contrast which highlights now only variations in atomic mass and not in thickness, facilitating thus the interpretation of the images in terms of spatial distributions of the chemical elements present in the specimen^24^. Electron tomography is the most appropriate technique for evaluating porosity characteristics at the nanoscale^25^. Applied to our specimens in their initial state, it shows that the crystal facets are quite regular and that the magnetosomes do not contain a nanoscale porosity inside, as shown by the typical slices extracted from the reconstructed volume (Fig. 4A–C). On the other hand, after the oxidation at 300 °C, the tomography results (Fig. 4D,E,F) show that the core of the crystals has not been modified; the corresponding volume is homogeneous and without porosity.

This result suggests that the magnetosome membrane prevents or slows down the O_2_ diffusion and thus protects the integrity of the stoichiometry and the structure of the magnetite at temperatures up to 300 °C.

Interestingly, high-resolution X-ray diffraction of magnetosomes and abiotic magnetite with similar size as magnetosomes has shown that biogenic nanomagnetite is stoichiometric^26^. The chemical removal of the magnetosome membrane in this study leads to the oxidation of magnetite, revealing the protective character of this biological membrane from oxidation^26^. To our knowledge, there is no detailed study that has characterized the biochemical properties associated with the protective effect of the magnetosome membrane against oxidation.

Severe damage or transformation was observed on the crystalline structure of the magnetite crystal and the membrane of magnetosomes when samples were heated to temperatures above 300 °C. Two typical areas on the E-chip containing magnetosomes were analysed. One of the areas has been continuously irradiated by the electron beam during the oxygen treatment (Fig. 5A–C); a clear morphological transformation of the irradiated magnetosomes can be observed which leads to aggregated amorphous chains at the end of the treatment. This combination of electron beam irradiation and heating effects to transform nanomaterials have been used to create nanoreactors in the field of the development of new nanometric structures^27^. The other areas of the E-chip containing magnetosome structures that were not irradiated during the heating process under oxidizing conditions (Fig. 5A,B) were observed in the “low dose” mode by reducing the dwell time and the spot size in STEM mode and adjusting the image focus and the electron dose in CTEM mode in a region far from the analysed area. This method is commonly used to study materials sensitive to radiation damage, as for instance biological materials and certain oxides^28^. In these conditions, by comparing the final morphologies from the two types of areas, continuously irradiated and irradiated only during the data acquisition in the low dose mode, we may unambiguously consider that the morphological and crystallographic transformations of the magnetite nanocrystals, characterized by the formation of hollow structures within the nanocrystals, is exclusively due to an oxidation process whose kinetics depends on the temperature and the speed of cation diffusion^18^.

In conclusion, as can be seen in Fig. 5C, when a region is observed continuously under irradiation, significant damage occurs, in particular, the aggregation of nanocrystals in the form of clusters. Therefore, damages observed in the non-irradiated crystals, which are represented by hollow structures or cavities, likely resulted from vacancy effect rather than damage from the electron beam. It is difficult to distinguish magnetite from the maghemite by electron diffraction and even by FFT of the high-resolution images (Supplementary Fig. S3). Indeed magnetite presents a mixed-valence given by the presence of Fe^2+^, Fe^3+ ^ions, shared between octahedral and tetrahedral sites, while maghemite (γ-Fe_2_O_3_) can be considered as oxidized magnetite, due to Fe^2+^oxidation, being composed only by Fe^3+^. Despite this, both have the cubic structure practically preserved, although in maghemite, the displacement of 11.1% of the iron creates vacancies that appear rather in the octahedral sites. The co-existing magnetite and maghemite resolution and identification are also complex due to the existence of a complete solid solution series between these two phases^20,21^. It has been shown that it is possible to distinguish iron oxidation states by EELS using a monochromatic (0.3 eV) electron source in an electron microscope^29^. The microscope used in this study does not allow us to determine this; the energetic resolution reached is at best 0.9 eV. Moreover, semi quantitative analysis (Fig. 6B,D) showed that the concentration of iron is higher outside the cavities, which makes it possible to conclude that there was a migration of iron along the magnetosome crystal. Gallagher *et al*.^18^ showed that the formation of maghemite is controlled by the diffusion of iron cations. Shidu *et al*.^21^, confirmed this mechanism by following the evolution of the content in Fe^2+ ^during oxidation and the formation of a hollow structure resulting of the diffusion of Fe^2+ ^cations. As has been showed in the case of metallic nanostructures, the oxidation process leads to the formation of a hollow structure through a nanoscale Kirkendall effect^30,31^. This phenomenon is explained by the differential of diffusion between the outward metal cations and the inward anions, such as oxygen. Their weaker effusivity is compensated by a flux of vacancies that condenses to form a hollow structure. In our case, a similar process is envisaged during the oxidation: diffusion of Fe^2+^outwards the magnetite with the inward diffusion of oxygen and vacancies that condense to form a hollow structure and formation of mixed oxides magnetite/maghemite on the surface of the nanocrystal of magnetite.

Considering the relevance of our results to paleomagnetism, the fact that magnetosome retains its intact structure up to 300 °C under oxidizing conditions, confirmed the contribution of magnetotactic microorganisms to the magnetization of sediments as well as a record of ancient ecosystems as proposed by Stolz *et al*.^32^. The [111] elongation direction of the crystal can be also determined by using the FFT from the high-resolution images (Fig. 1G). Pósfai *et al*.^33^ discussed that elongation of magnetosomes in magnetotactic bacteria is parallel to the [111] crystal axis for the equidimensional (cuboctahedral and octahedral magnetosomes) and elongated-prismatic shapes, while the elongation axes in anisotropic magnetosomes could be <100>, <110>, or <111>. The easy axis of magnetite magnetization is parallel to <111> axis. Thus, magnetite magnetosomes that present this characteristic can be considered the most evolved from the evolutionary point of view^33^. <111> is also the elongation axis of most magnetite mineralized abiotically; however, crystal morphology and dimensions of these particles are not similar to those biomineralized by magnetotactic bacteria. Elongation axis, shape and size of each type of magnetosome seem to be related to the evolution of magnetotactic bacteria and are considered as a signature of biogenic magnetite^33^. According to our results, exposure to temperatures of approximately 500 °C partially transforms the crystalline structure of magnetosomes. However, after this treatment, the obtained hollow structure partially retains the memory of the original magnetite structure as shown by its elongated morphology and the associated FFT (Fig. 5D,E), which can be interpreted as resulting from magnetite or maghemite because their structures are very close. Indeed, this transformation is a topotactic transformation that retains the starting structure^34^.

It is important to note that, to definitively confirm the oxidation of magnetite in maghemite, the quantitative use of other more macroscopic techniques such as *ex situ* synchrotron X-ray diffraction associated with magnetometry techniques and Mossbauer spectroscopy is mandatory, but these analyses require a large amount of magnetosomes.

Therefore, our results showed that even if a magnetofossil has been subjected to extreme conditions, for example, entrance in Earth’s atmosphere, it might be possible to predict its biological origin, since crystallographic information is resistant to high temperatures, even if localized damaged caused by heat, as cavities observed in this study occurs. Thomas-Kepra *et al*.^35^ reported the presence of magnetite nanocrystals resembling magnetosomes in the Martian ALH84001 meteorite and suggested the existence of carbonate globules and rims in the meteorite, which might have protected magnetofossil from damage. Results presented here probably will help researchers in identifying magnetofossils damaged by high temperatures in relevant specimens, contributing to future analysis of extra-terrestrial or extreme environments fossil record.

From the biotechnological point of view, our results showed that magnetosomes can be subjected to high temperature and still maintain their structural and chemical characteristics, and, consequently, their magnetic properties. In nanomedicine, temperatures higher than 100 °C will probably not be applied, thus the use of magnetosomes as thermo-controlled drug-delivery systems, as proposed by Santos *et al*.^36^ with artificially synthesized superparamagnetic iron oxide nanoparticles, and magnetic hyperthermia probably would avoid the need of constant administration of the nano-formulation in the organism during treatment. The benefit of resistance to temperatures between 300 and 500 °C is directly related to the industrial application of magnetosomes to the recovery of molecules or metals and in nanobiocatalysis processes. Yoshino *et al*.^37^ developed a thermoresponsive magnetosome in which the elastin-like polypeptide was expressed in the surface of the magnetosome. At temperatures above 60 °C, the hydrophobicity of these nanoparticles increased, promoting their aggregation. According to our results, temperature up to 300 °C would be safe to maintain the magnetosome structure, and thus guarantee easy recovery and reuse of this biotechnological tool. Magnetosomes usage in biotechnological application is considered advantageous over artificially synthesized magnetite nanocrystals, because of their easy and effective functionalization, relatively cheap and ecologically-safe production, unique magnetic properties, narrow size and shape distribution and low aggregation between particles due to the presence of the bilipid layer. Thus, the results presented in this study showed the good thermal stability of magnetosomes and promising application in the development of stable nano-enzymatic complexes. Possibly, magnetosomes could be used in the improvement of nano-enzymatic complexes developed by the immobilization of structurally-stable hydrolases onto artificially synthesized nanoparticles that showed imprecise results regarding efficiency stability and reuse^38^.

In summary, the results presented here define a temperature range up to 300 °C as safe to maintain magnetosome structure preservation, which would ensure easy retrieval and reuse of this biotechnological tool in industrial applications that employ high temperatures. In addition, this work exhibits the employment of ground-breaking nanoanalytical *in situ* and 3D-TEM techniques for characterization studies of biogenic nanomaterials.

## Materials and methods

### Cell cultivation and magnetosome purification

Cells of *Magnetovibrio blakemorei* strain MV-1 were grown in a 5 L bioreactor (Minifors, Infors HT, Basel, Switzerland) as described previously^39^. After 192 h of incubation, cells were collected by centrifugation (7,000 x g) and resuspended in 50 mL HEPES buffer (10 mM; pH 6.8). Cells were then disrupted by sonication (VCX 500, Sonics, Newtown, USA) at 20% amplitude and 20 kHz frequency for 1 h (60 cycles of 30 s between intervals of 30 s). Magnetosomes were magnetically concentrated for 12 h at 4 °C with a neodymium-boron magnet attached to the bottom of the tube containing the magnetosomes. The supernatant was replaced by HEPES buffer (10 mM) with NaCl (200 mM), washed 4 times in a bench sonicator (Branson 2200) for 30 min (each washing step) and concentrated again using the same magnet. Finally, supernatant was discarded and the magnetosomes resuspended in distilled, deionized H_2_O (1 mL).

### Transmission electron microscopy and *in situ* thermal stability analysis

Magnetosome purification was verified by depositing magnetosome suspension (10 μL) directly on a formvar-coated copper grid (Electron Microscopy Sciences, USA), and observing this sample in a Morgagni transmission electron microscope (FEI Company, Hillsboro, OR, USA) with an acceleration voltage of 80 kV.

For the thermal stability *in situ* evaluation, the extracted magnetosome suspension (1 μL) was deposited on a Si_3_N_4_ membrane of the E-chip used for the environmental gas analysis and air-dried for 1 h. The *in-situ* experiments observations were carried out using a Protochips Atmosphere Gas Cell device. This later allows to heat the sample under controlled gas flow in the gas cell device. All the indicated temperatures are based on the company provided calibration. The microscope used for samples examination was a Jeol 2100 F FEG equipped with a spherical aberration corrector, operating at 200 kV in CTEM and in the BF-STEM or HAADF-STEM modes with a resolution of 0.11 nm. Three types of experiments were carried out using the conventional TEM (CTEM), scanning TEM(STEM) and STEM tomography. Initially, argon gas (1 atm pressure) was inserted into the *in situ* chip chamber and the temperature was raised to 150 °C. After 1 h, the Ar was replaced by O_2_ (1 atm pressure) and the samples were exposed to the following temperatures: 150, 200, 300 and 500 °C. During the experiment various CTEM images were systematically acquired under controlled irradiation conditions to evaluate the effect of the electron beam irradiation on the magnetosome structure. Finally, at 500 °C in the areas corresponding to the periodically irradiated region, high-resolution images were acquired. All images were obtained while the sample was at high temperature, excepting those acquired simultaneously to the energy-dispersive X-ray spectroscopy (EDS) analysis. For EDS measurement, we removed the silicon nitride window of the holder’s chamber to maximize the signal to noise ratio in the spectra by increasing the amount of X-rays emitted by the sample and acquired by the detector. A Silicon Drift Detector (SDD-EDS) was used to obtain X-ray (EDS) and a Gatan Imaging Filter (GIF) (Gatan Inc., Pleasanton, CA, USA) with 0.9 eV energy resolution was used to obtain EELS spectra. Digital Micrograph software (Gatan Inc., Pleasanton, CA, USA) and JEMS software (http://www.jems-saas.ch/) were used to quantitatively analyse the images. The tomography was performed in HAADF-STEM mode by considering the angular range from −55° to +55° with a step of 1.5° between two successive images.

## Supplementary information

Supplementary Information.

## References

[1] Bazylinski DA, Frankel RB (2004). Magnetosome formation in prokaryotes. Nature Rev. Microbiol..

[2] Uebe R, Schüler D (2016). Magnetosome biogenesis in magnetotactic bacteria. Nature Rev. Microbiol..

[3] Lin W (2017). Origin of microbial biomineralization and magnetotaxis during the Archean. Proc. Natl. Acad. Sci. USA.

[4] Kopp RE, Kirschvink JL (2008). The identification and biogeochemical interpretation of fossil magnetotactic bacteria. Earth-Sci. Rev..

[5] Chang SR, Kirschvink JL (1989). Magnetofossils, the magnetization of sediments, and the evolution of magnetite biomineralization. Annu. Rev. Earth and Planet Sci..

[6] Jovane L, Florindo F, Bazylinski DA, Lins U (2012). Prismatic magnetite magnetosomes from cultivated *Magnetovibrio blakemorei* strain MV-1: a magnetic fingerprint in marine sediments. Environ. Microbiol. Rep..

[7] Kopp RE (2007). Magnetofossil spike during the Paleocene-Eocene thermal maximum: Ferromagnetic resonance, rock magnetic, and electron microscopy evidence from Ancora, New Jersey, United States. Paleoceanography.

[8] Savian JF (2016). Environmental magnetic implications of magnetofossil occurrence during the Middle Eocene Climatic Optimum (MECO) in pelagic sediments from the equatorial Indian Ocean. Palaeogeogr Palaeoclimatol Palaeoecol..

[9] Abraçado LG (2014). Ferromagnetic resonance of intact cells and isolated crystals from cultured and uncultured magnetite-producing magnetotactic bacteria. Phys. Biol..

[10] Dunin-Borkowski RE (2001). Off-axis electron holography of magnetotactic bacteria: Magnetic microstructure of strains MV-1 and MS-1. Eur. J. Mineral..

[11] Werckmann J (2017). Localized iron accumulation precedes nucleation and growth of magnetite crystals in magnetotactic bacteria. Sci. Rep..

[12] Vargas G (2018). Applications of magnetotactic bacteria, magnetosomes and magnetosome crystals in biotechnology and nanotechnology: mini-review. Molecules.

[13] Sun JB (2008). Preparation and anti‐tumor efficiency evaluation of doxorubicin‐loaded bacterial magnetosomes: Magnetic nanoparticles as drug carriers isolated from *Magnetospirillum gryphiswaldense*. Biotechnol. Bioeng..

[14] Alphandéry E, Faure S, Seksek O, Guyot F, Chebbi I (2011). Chains of magnetosomes extracted from AMB-1 magnetotactic bacteria for application in alternative magnetic field cancer therapy. ACS nano.

[15] Xu J (2014). Surface expression of protein A on magnetosomes and capture of pathogenic bacteria by magnetosome/antibody complexes. Front. Microbiol..

[16] Honda T, Tanaka T, Yoshino T (2015). Stoichiometrically controlled immobilization of multiple enzymes on magnetic nanoparticles by the magnetosome display system for efficient cellulose hydrolysis. Biomacromolecules.

[17] Liu RT (2012). Heating effect and biocompatibility of bacterial magnetosomes as potential materials used in magnetic fluid hyperthermia. Pro. Nat. Sci-Mater.

[18] Gallagher KJ, Feitknecht W, Mannweiler U (1968). Mechanism of oxidation of Magnetite to γ-Fe_2_0_3_. Nature.

[19] Colombo U (1968). Mechanism of low temperature oxidation of magnetites. Nature.

[20] Topsøe H, Dumesic J, Boudart M (1974). Mössbauer Spectra of Stoichiometric and Nonstoechiometric Fe_3_O_4_ Microcrystals. J. Phys. Colloq..

[21] Sidhu PS, Gilkes RJ, Posner AM (1977). Mechanism of the low temperature oxydation of synthetic magnetites. J. Inorg. Nucl. Chem..

[22] Lewis RNAH, McElhaney RN (2013). Membrane lipid phase transitions and phase organization studied by Fourier transform infrared spectroscopy. Biochim. Biophys. Acta- Biomembr..

[23] Nellist PD, Pennycook SJ (2000). The principles and interpretation of annular Dark-Field Z-contrast imaging. Advances in Imaging and Electron Physics.

[24] Arenal R, Roiban L, Ersen O, Burgin J, Treger-Delapierre M (2011). Gold Nanoparticles: 3D-STEM-HAADF Analyses and Plasmonic Studies by EELS. Microsc. Microanal..

[25] Florea I (2011). 3D-TEM characterization of the porosity in nanoscaled materials: Application to catalysis. Adv. Eng. Mater..

[26] Fischer A, Schmitz M, Aichmayer B, Fratzl P, Faivre D (2011). Structural purity of magnetite nanoparticles in magnetotactic bacteria. J. R. Soc. Interface.

[27] Krasheninnikov AV, Banhart F (2007). Engineering of nanostructured carbon materials with electron or ion beams. Nat. Mater..

[28] Williams, D. & Carter, C. Transmission Electron Microscopy: A Textbook forMaterials Science. (ed. Springer) (2009).

[29] Almeida TP (1974). Visualized effect of oxidation on magnetic recording fidelity in pseudo-single-domain magnetite particles. Nat. Commun..

[30] Yin Y (2004). Formation of Hollow Nanocrystals Through the Nanoscale Kirkendall Effect. Science.

[31] Railsback JG, Johnston-Peck AC, Wang J, Tracy JB (2010). Size-Dependent Nanoscale Kirkendall Effect During the Oxidation of Nickel Nanoparticles. ACS Nano.

[32] Stolz JF, Lovley DR, Haggerty SE (1990). Biogenic magnetite and the magnetization of sediments. J. Geophys. Res..

[33] Pósfai M, Lefèvre CT, Trubitsyn D, Bazylinski DA, Frankel RB (2013). Phylogenetic significance of composition and crystal morphology of magnetosome minerals. Front. Microbiol..

[34] Cudennec Y, Lecerf A (2005). Topotactic Transformations of Goethite and Lepidocrocite into Hematite and Maghemite. Solid State Sci..

[35] Thomas-Keprta KL (2000). Elongated prismatic magnetite crystals in ALH84001 carbonate globules: potential Martian magnetofossils. Geochim. Cosmochim. Acta..

[36] Santos ECS (2018). AMF-responsive doxorubicin loaded β-cyclodextrin-decorated superparamagnetic nanoparticles. New J. Chem..

[37] Yoshino T (2018). Biosynthesis of Thermoresponsive Magnetic Nanoparticles by Magnetosome Display System. Bioconjugate Chem..

[38] Kumari A (2018). Multiple thermostable enzyme hydrolases on magnetic nanoparticles: An immobilized enzyme-mediated approach to saccharification through simultaneous xylanase, cellulase and amylolytic glucanotransferase action. Int. J. Biol. Macromol..

[39] Silva KT (2013). Optimization of magnetosome production and growth by the magnetotactic vibrio *Magnetovibrio blakemorei* strain MV-1 through a statistics-based experimental design. J. Appl. Environ. Microbiol..

