# Supplementary material for: Insight on thermal stability of magnetite magnetosomes: implications for the fossil record and biotechnology
Source: Sci Rep. 2020 Apr 21;10:6706. doi: 10.1038/s41598-020-63531-5 (PMC7174351; doi:10.1038/s41598-020-63531-5)
Supplement: Supplementary file 1 — Supplementary Information. [file 41598_2020_63531_MOESM1_ESM.docx]

**Insight on thermal stability of magnetite magnetosomes: implications for the fossil record and biotechnology**

Jefferson Cypriano^1^, Mounib Bahri^2^, Kassiogé Dembelé^2’6^, Walid Baaziz^2^, Pedro Leão^1^, Dennis A. Bazylinski^3^, Fernanda Abreu^1^, Ovidiu Ersen^2^, Marcos Farina^4^ and Jacques Werckmann^4,5*^

^1^ Instituto de Microbiologia Paulo de Góes, Universidade Federal do Rio de Janeiro, Brazil

^2^ Institut De Physique et Chimie Des Matériaux De Strasbourg, Strasbourg (IPCMS), UMR 7504 CNRS-Université de Strasbourg 23 rue du Loess 67034 Strasbourg France

^3^ School of Life Sciences, University of Nevada at Las Vegas, Las Vegas, 89154-4004, USA

^4^ Instituto de Ciências Biomédicas, Universidade Federal do Rio de Janeiro, Brazil

^5^ Centro Brasileiro de Pesquisas Físicas, rua Xavier Sigaud, 150, CEP 22290-180 Rio de Janeiro, Brazil

^6^ Fritz-Haber-Institut der Max-Planck-Gesellschaft, Department of Inorganic Chemistry,

Faradayweg 4-6, 14195 Berlin Germany

*** Corresponding author**

**E-mail:** j.werckmann@gmail.com

**Supplementary Figures**


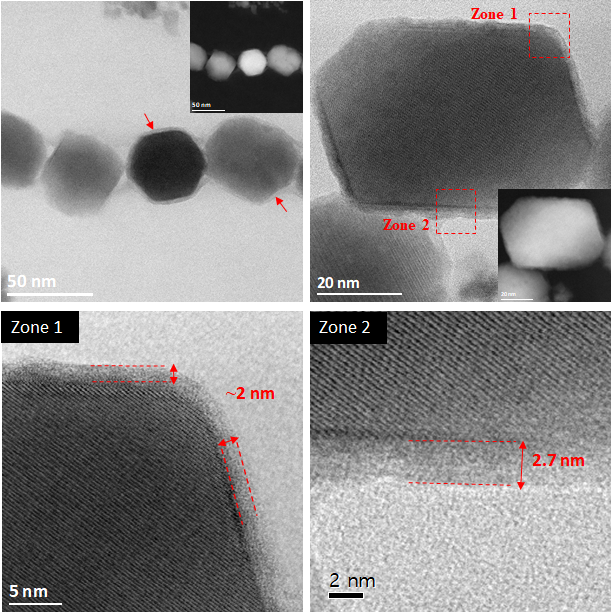


**Figure S1.** BF-STEM images of magnetosome before oxidation in the box corresponding HAADF images. (A) Part of magnetosome chain, red arrows highlight the presence of the bilipidic membrane. (B) Individual crystal of magnetite zone 1 and zone 2 where magnified image (C) and (D). (C) Magnification of the zone 1, thickness of the membrane of about 2 nm. (D) Magnification of the zone 2, thickness of the membrane of about 2.7 nm.


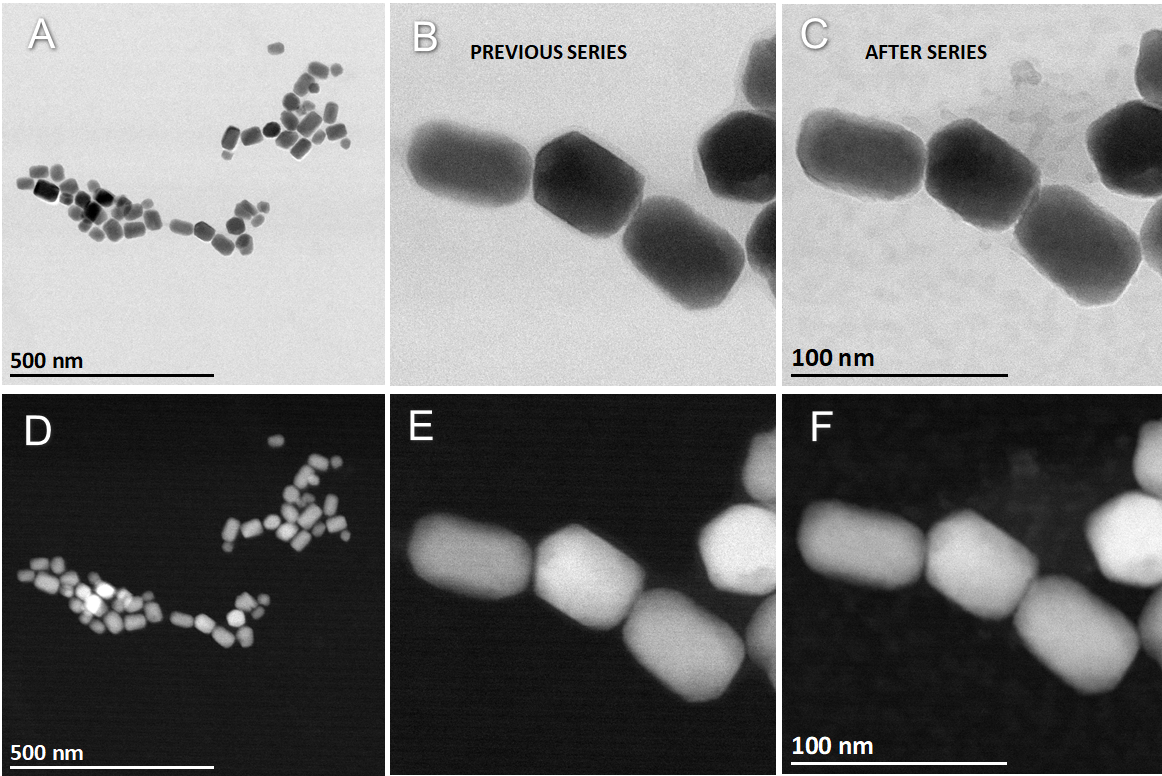


**Figure S2.** BF-STEM and HAADF-STEM images. (A) (B) (D) (E) images acquired before, (C) and (F) after the tomography before oxidation, without the sealing cover.


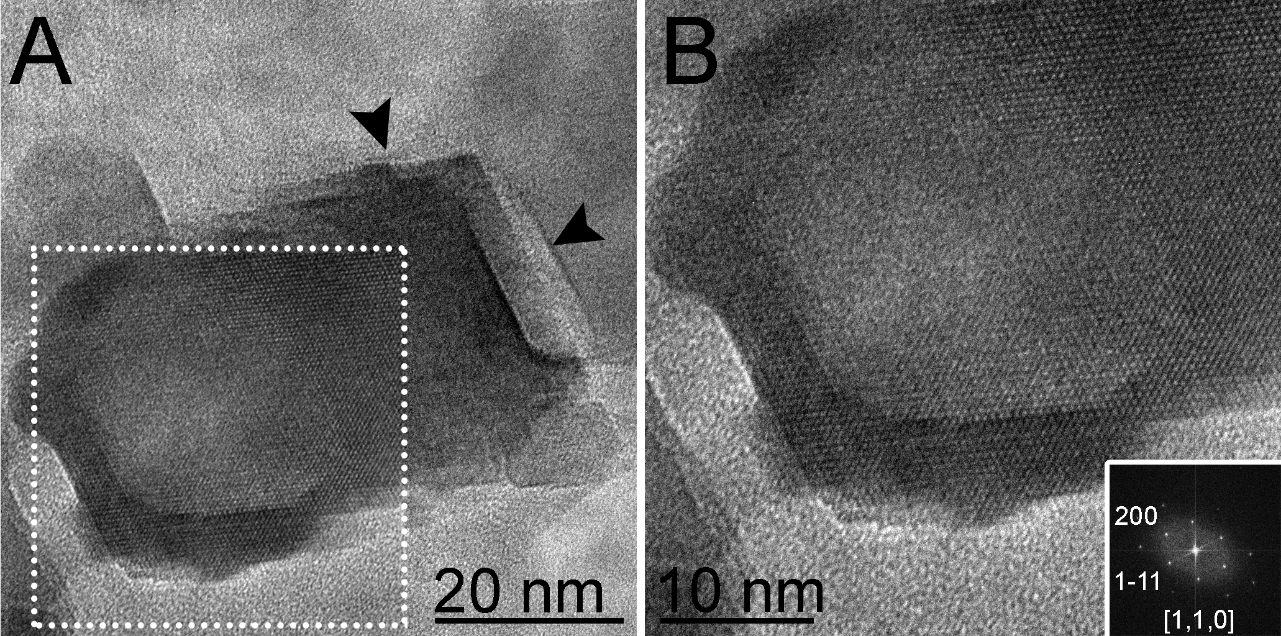


**Figure S3.** High-resolution CTEM images of magnetosome heated to 500 °C in the *in situ* experiment with O_2_. (A) Magnetosome showing a large cavity (area delimited by the white dashed square) and border defects (arrowheads); (B) Higher magnification of magnetosome displayed in (A) showing the damaged crystalline structure and irregular facets in detail. The inset shows an FFT of image in (B) with indexed planes consistent with theoretical angles and distances expected for magnetite or maghemite, based on magnetite cubic pattern with 8.396Ǻ unit cell parameter and structure of maghemite derived from magnetite structure.

**
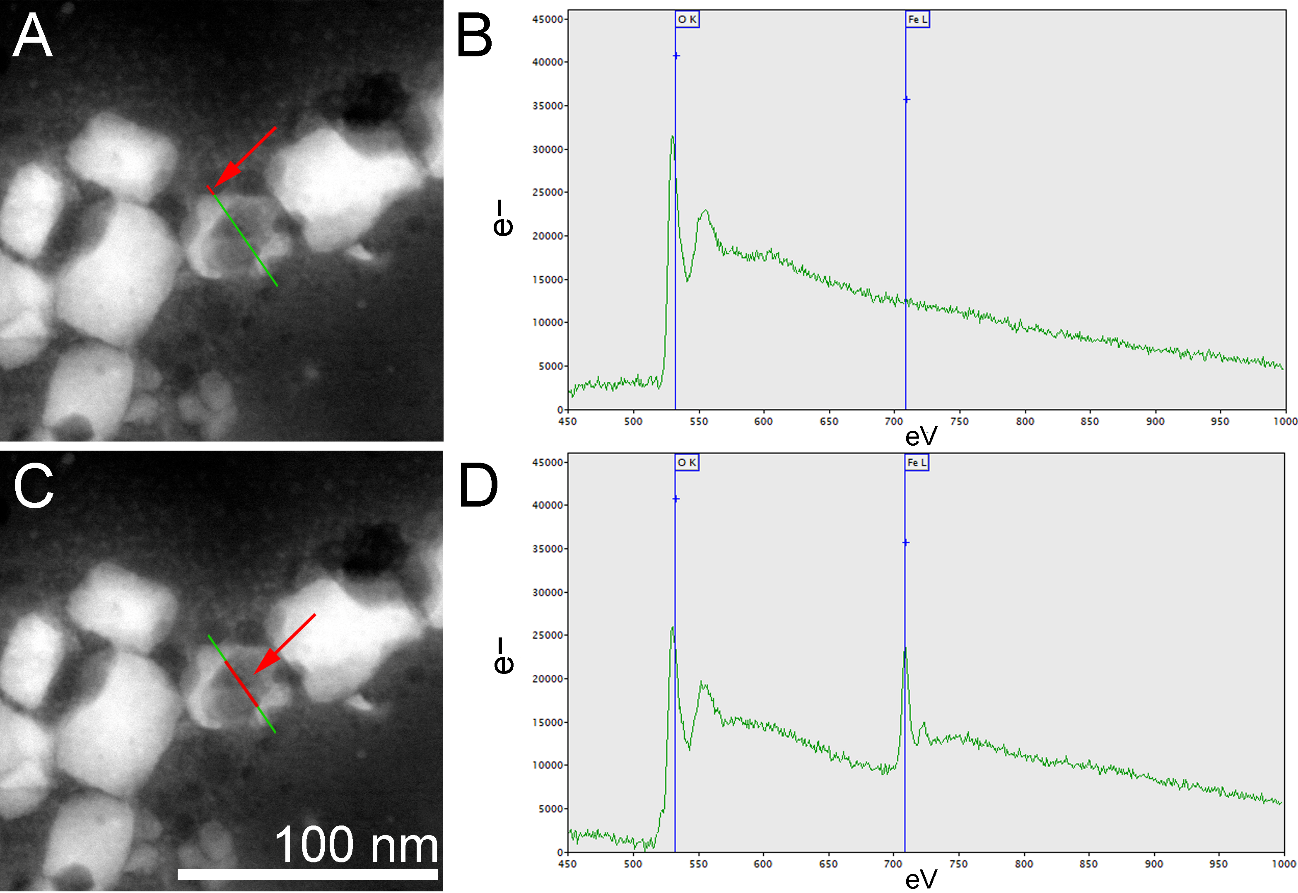
**

**Figure S4.** HAADF-STEM images and EELS microanalysis of magnetosomes after heating until 500 °C and exposure to O_2_, showing the irregular contrast on magnetosomes and the presence of large cavities. (A) STEM image of magnetosomes showing the region outside crystal used in EELS analysis (red line); (B) EELS spectra of the region displayed in (A) showing the oxygen peak at 540 eV and a smaller iron peak at 710 eV; (C) STEM image of magnetosomes showing the region of a cavity in the crystal used in EELS analysis (red line); (D) EELS spectra of the region displayed in (C) showing a large oxygen peak at 540 eV and iron peaks 710 eV. Scale bar in (C) applies to (A).


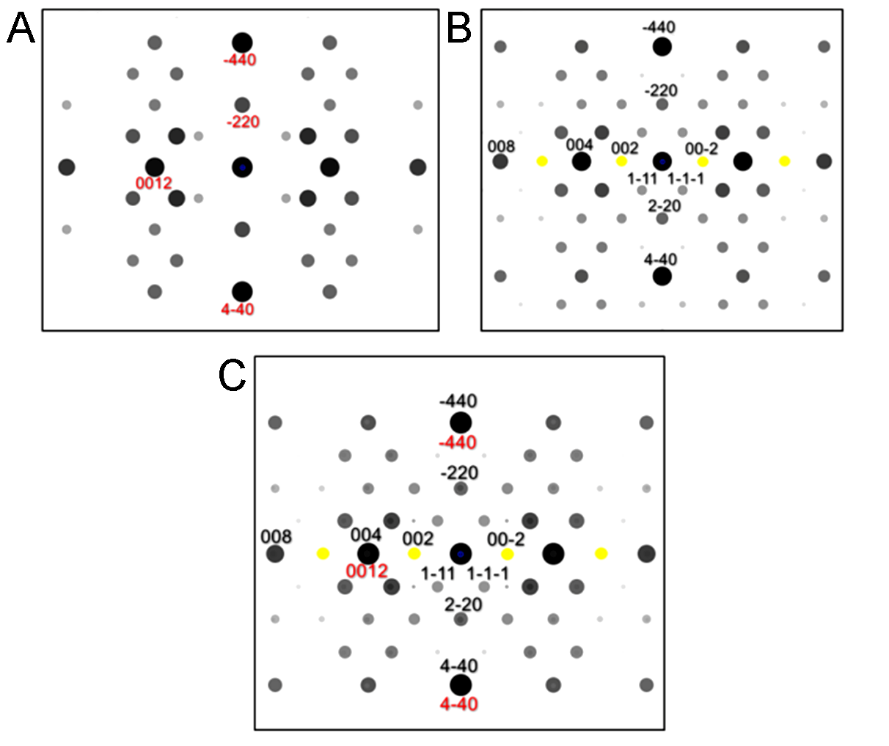


**Figure S5.** Diffraction patterns simulation of maghemite and magnetite in the [110] zone axis direction: (A) Maghemite; (B) Magnetite with the double diffractions characteristic of a perfect crystal shown in yellow; (C) Superposition of the diffraction pattern, in yellow double diffraction from magnetite and the indexation corresponding respectively to magnetite in black to the maghemite in red. The oxidation reaction of magnetite to maghemite is a topotactic reaction, the oxygen network is conserved, so the [110] zone axis of maghemite match exactly with the [110] zone axis of magnetite.
